# Circulating levels of sphingosine-1-phosphate are elevated in severe, but not mild psoriasis and are unresponsive to anti-TNF-α treatment

**DOI:** 10.1038/srep12017

**Published:** 2015-07-15

**Authors:** Antonio Checa, Ning Xu, Daniel G. Sar, Jesper Z. Haeggström, Mona Ståhle, Craig E. Wheelock

**Affiliations:** 1 Department of Medical Biochemistry and Biophysics, Division of Physiological Chemistry 2, Karolinska Institutet, SE-17177, Stockholm, Sweden; 2 Dermatology Unit, Department of Medicine, Karolinska Institutet, SE-17176, Stockholm, Sweden

## Abstract

Sphingolipids are bioactive molecules with a putative role in inflammation. Alterations in sphingolipids, in particular ceramides, have been consistently observed in psoriatic skin. Herein, we quantified the circulating sphingolipid profile in individuals with mild or severe psoriasis as well as healthy controls. In addition, the effects of anti-TNF-α treatment were determined. Levels of sphingoid bases, including sphingosine-1-phosphate (S1P), increased in severe (*P* < 0.001; n = 32), but not in mild (n = 32), psoriasis relative to healthy controls (n = 32). These alterations were not reversed in severe patients (n = 16) after anti-TNF-α treatment despite significant improvement in psoriasis lesions. Circulating levels of sphingomyelins and ceramides shifted in a fatty acid chain length-dependent manner. These alterations were also observed in psoriasis skin lesions and were associated with changes in mRNA levels of ceramide synthases. The lack of S1P response to treatment may have pathobiological implications due to its close relation to the vascular and immune systems. In particular, increased levels of sphingolipids and especially S1P in severe psoriasis patients requiring biological treatment may potentially be associated with cardiovascular comorbidities. The fact that shifts in S1P levels were not ameliorated by anti-TNF-α treatment, despite improvements in the skin lesions, further supports targeting S1P receptors as therapy for severe psoriasis.

Psoriasis is a common chronic inflammatory skin disease, which affects nearly 3% of the world’s population. The characteristic pathological changes of psoriatic skin lesions are hyperproliferation and deregulated differentiation of epidermal keratinocytes and massive infiltration of immune cells into the skin^1^. Severe psoriasis is a systemic disease with significant co-morbidities, such as psoriatic arthritis and metabolic syndrome^2^. In recent years, biological therapies including anti-tumor necrosis factor (TNF)-α treatment have greatly increased therapeutic choices for patients with severe psoriasis. In addition to their efficacy, the use of targeted immune antagonists offers the opportunity to study the contribution of specific immune molecules and pathways in psoriasis pathology^1^.

Sphingolipids are not only eukaryotic-specific cell membrane lipids, but also perform important intracellular and extracellular functions and are recognized as critical regulators in human physiology and pathology^3^. Ceramides are the central hub of the sphingolipid pathway (Supplementary Figure 1). In the skin, together with free fatty acids and cholesterol, ceramides are the main lipids in stratum corneum^4^, and are responsible for maintaining the water permeability barrier in a fatty-acyl chain length dependent manner^5^. In accordance with this, recent work has suggested that fatty acids linked to ceramides in psoriatic lesions have shorter chain lengths^6^.

Sphingosine-1-phosphate (S1P) is a pleiotropic bioactive sphingolipid formed by the action of sphingosine kinases inside cells. S1P has attracted attention for its effects on keratinocytes driving differentiation and suppressing proliferation^7^. In addition, extracellular S1P has been thoroughly studied regarding its effects on immune cell trafficking and on the vascular system (*e.g.,* permeability, angiogenesis, atherosclerosis and heart functions)^8^. While multiple studies have examined the dysregulation of sphingolipids in psoriatic skin, little is known about their circulatory levels. Accordingly, in the current study we compared circulating sphingolipid levels in healthy controls to individuals with mild and severe psoriasis to profile these bioactive lipids in disease and test the hypothesis that circulating sphingolipids are increased in severe psoriasis. In particular, we wanted to further examine our previously reported alterations in the sphingolipid pathway observed in patients with severe psoriasis^9^. We focused on the non-hydroxylated fatty acid/sphingosine (NS) sphingolipids because they are the most abundant class in circulation, and we also quantified their levels in skin biopsies. In addition, the effects of anti-TNF-α treatment upon sphingolipid metabolism, and specifically S1P levels, in severe psoriasis patients were examined.

## Results

### Levels of plasma sphingoid bases were higher in individuals with severe psoriasis relative to mild psoriasis and healthy controls

**Figure 1:**
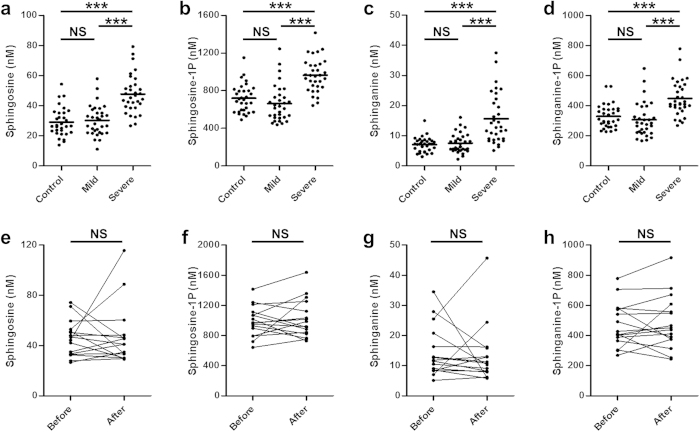
Circulating sphingoid bases are increased in severe psoriasis patients. (**a**–**d**) Plasma levels of sphingoid bases are increased in severe psoriasis patients (n = 32) in comparison to healthy controls (n = 32) and mild psoriasis patients (n = 32). Each point represents an individual. A horizontal line shows the mean value for the group. (**e**–**h**) Levels of sphingoid bases are not affected by anti-TNF-α treatment in severe psoriasis patients (n = 16). Connected dots represent one individual. Normality was assessed by the Kolmogorov-Smirnov test. The statistical significance for multiple group comparisons was determined via a one way ANOVA with Tukey’s post-hoc correction. Pairwise comparisons were performed using the Wilcoxon signed-rank test. ***P < 0.001, NS = Not significant.

Using ultra performance liquid chromatography—tandem mass spectrometry (UPLC-MS/MS), we quantified the sphingolipid levels in the plasma of patients with mild (n = 32) or severe psoriasis (n = 32) and healthy donors (n = 32) (Table 1). In addition, levels of circulating sphingolipids were determined in 16 of the severe psoriasis patients after 12 weeks of treatment with the anti-TNF-α drug Etanercept. Sphingolipids are discussed in terms of the lipid class (*e.g,* hexosylceramides) and the associated fatty acid chain (*e.g.,* palmitic acid). The fatty acid nomenclature depends upon the length of the alkyl chain and degree of unsaturation. For example, lauric acid contains a 12 carbon saturated alkyl chain (C_12:0_) and nervonic acid possesses a 24 carbon alkyl chain with a single double bond (C_24:1_). Because of their high abundance in plasma, our analysis focused on the NS class of sphingolipids. In addition, NS is one of only two sphingomyelin classes that can produce ceramides by hydrolysis in the stratum corneum. Our analysis included extensive coverage of the sphingolipid pathway (30 species in total were quantified), consisting of a range of compounds including sphingomyelins, ceramides, hexosylceramides, lactosylceramides and dihydroceramides with varying fatty acid chain lengths (Supplementary Table 1). The analysis also included free phosphorylated and non-phosphorylated NS sphingoid bases (sphingosine, sphinganine, S1P and sphinganine-1-phosphate [Spa1P]). Supplementary Figure 1 provides an overview of sphingolipid metabolism. Circulating levels of sphingosine, S1P, sphinganine and Spa1P were significantly elevated (*P* < 0.001) in severe psoriasis patients compared with healthy controls and mild psoriasis groups. There were no significant differences between the healthy individuals and patients with mild psoriasis (Fig. 1a–d).

### Anti-TNF therapy did not normalize sphingoid bases levels

As expected, patients responded to Etanercept treatment with a significant improvement in psoriatic lesions as reflected by the PASI score (*P* < 0.001; Table 1). However, circulating levels of sphingoid bases levels in severe psoriasis patients were not affected by treatment (Fig. 1e–h).

### Circulating levels of ceramides and sphingomyelins showed fatty acid chain length-dependent alterations in association with disease severity

**Figure 2:**
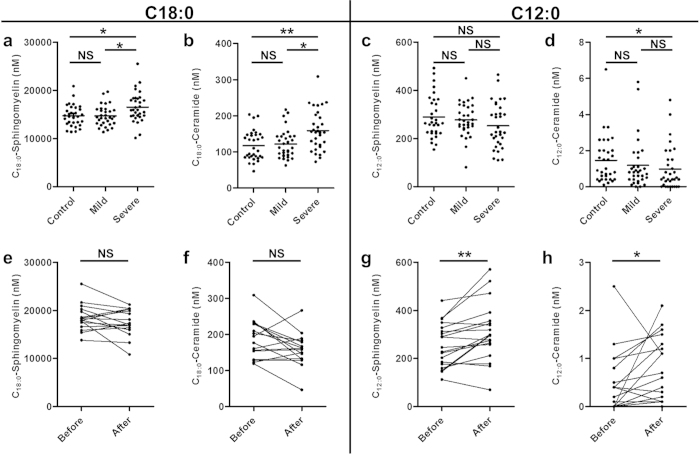
Levels of circulating ceramides evidence fatty acyl chain length-dependent shifts. (**a**–**d**) Plasma levels of sphingomyelins and ceramides are increased in severe psoriasis patients (n = 32) in comparison to healthy controls (n = 32) and mild psoriasis patients (n = 32) for C_18:0_ fatty acid chain-containing analogs, whereas the levels of C_12:0_ fatty acid chain-containing ceramides are decreased. Each point represents an individual. A horizontal line shows the mean value for the group. (**e**–**f**) Anti-TNF-α treatment (n = 16) in severe psoriasis patients resulted in increased C_12:0_ fatty acid chain-containing sphingolipids and did not affect levels of C_18:0_ fatty acid chain-containing sphingolipids. Connected dots represent one individual. Statistical significance for multiple comparisons was determined via a Kruskal-Wallis test with Dunn´s post-hoc correction. Pairwise comparisons were performed using the Wilcoxon signed-rank test. C_12:0_ = fatty acid chain with 12 carbons (lauric acid); C_18:0_ = fatty acid chain with 18 carbons (stearic acid). *P < 0.05, **P < 0.01, NS = Not significant.

Elevated levels (*P* < 0.05) were observed for most analyzed ceramides (C_16:0_, C_18:0_, C_20:0_, C_22:0_ and C_24:1_) in severe patients *vs.* healthy controls (Supplementary Table 1). In the case of the C_18:0_ chain length, increases were also observed for the sphingomyelin and ceramide species (Fig. 2a,b). Similarly to the sphingoid bases, increases in the circulating levels of these compounds were not ameliorated following Etanercept treatment (Fig. 2e,f, Supplementary Table 2). Shorter fatty acid chain length sphingolipids exhibited a different pattern, with no changes in C_14:0_-ceramide and a potential trend towards decreased levels of C_12:0_-sphingomyelin in severe psoriasis patients *vs.* healthy controls (Fig. 2c). C_12:0_-ceramide was the only compound that decreased in severe psoriasis relative to healthy controls (Fig. 2d). No shifts were observed in the levels of the hexosylceramides, lactosylceramides or the remainder of the analyzed sphingomyelins (Supplementary Table 1). Following Etanercept treatment, levels of C_12:0_-sphingolipids increased, with significant increases observed for C_12:0_-sphingomyelin (*P* = 0.004; Fig. 2g), C_12:0_-ceramide (*P* = 0.035; Fig. 2h) and C_12:0_-lactosylceramide (*P* = 0.038; Supplementary Table 2). No differences in circulating sphingolipid levels were observed between the healthy controls and mild psoriasis groups.

### Sphingolipid profiles were altered in the skin of psoriasis patients in a fatty acid chain length-dependent manner

**Figure 3:**
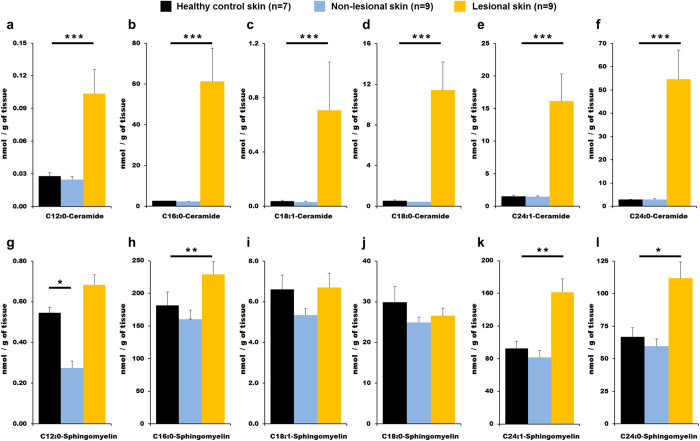
Levels of ceramides and sphingomyelins in lesional and non-lesional skin from severe psoriasis patients compared to healthy controls. Data are presented as the mean ± SEM. Statistical significance was determined via a Kruskal-Wallis test with Dunn´s post-hoc correction comparing to the healthy control group. *P < 0.05; **P < 0.01; ***P < 0.001.

Levels of sphingolipids were determined in lesional and non-lesional skin from severe psoriasis patients (n = 9), taken from a different cohort than the analyses for circulating species, and compared to those of healthy controls (n = 7). Increased levels (*P* < 0.001) for all ceramides were observed in lesional skin relative to non-lesional and control skin (Fig. 3a–f, Supplementary Figure 2). Levels of sphingomyelins were altered in lesional skin in a fatty acid chain length-dependent manner (Fig. 3g–k), with increases in C_16:0_-, C_24:1_- and C_24:0_-sphingomyelins. Levels of C_12:0_-sphingomyelin were lower in non-lesional skin *vs.* lesional and control skin (Fig. 3g). Results for the remainder of the compounds are presented in Supplementary Figure 2 and show increases in the levels of sphingosine and sphinganine as well as lactosylceramides and dihydroceramides in psoriasis lesional skin.

### Expression levels of enzymes in the sphingolipid biosynthetic pathway shifted in lesional skin

**Figure 4:**
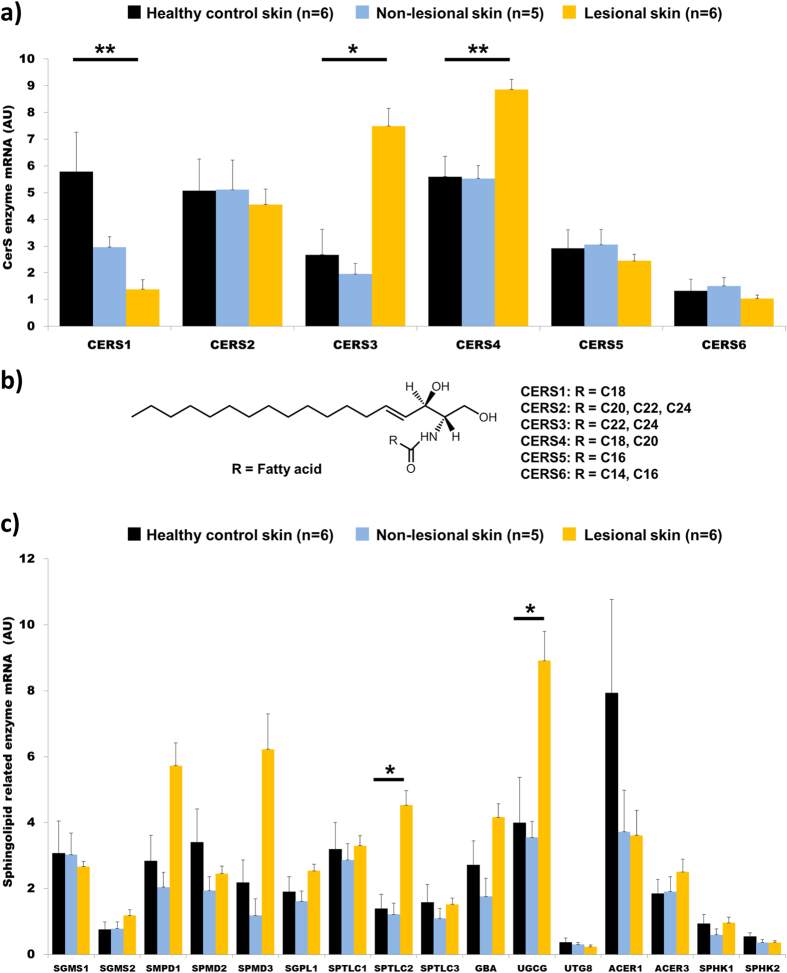
mRNA levels of ceramide synthases (CerS) and other sphingolipid pathway related enzymes are altered in lesional relative to non-lesional skin from severe psoriasis patients and healthy controls. (**a**) Levels of CerS in the different groups. (**b**) Structure of ceramide and affinities of CerS for different fatty acyl chain lengths included in the present study (Adapted from Levy and Futerman, 2010^33^). (**c**) Levels of other sphingolipid pathway related enzymes. Data are presented as mean ± SEM. AU = Arbitrary units normalized based on the expression of the internal positive control 18S RNA. One extreme outlier was excluded from the non-lesional skin group. Statistical significance was determined via a Kruskal-Wallis test with Dunn´s post-hoc correction comparing to the healthy control group. The enzyme nomenclature is as defined in the Materials and Methods. *P < 0.05; **P < 0.01.

The levels of the 6 known ceramide synthases (CerS) and other sphingolipid pathway-related enzymes were measured in lesional and non-lesional skin from severe psoriasis patients (n = 6) and compared to the levels present in skin from healthy controls (n = 6). A number of shifts were observed in lesional skin, with decreases in CerS1 (*P* < 0.01) and increases in CerS3 (*P* < 0.05) as well as CerS4 (*P* < 0.01) (Fig. 4a). The analysis of 16 additional enzymes involved in the sphingolipid biosynthetic pathway only evidenced increases in Serine Palmitoyltransferase Long-Chain 2 (SPTLC2) and UDP Glucose Ceramide Glucosyltransferase (UGCG) in lesional skin (Fig. 4c).

## Discussion

Sphingolipids are a complex lipid mediator family with both structural and signaling functions that are crucial in skin barrier formation and maintenance^10^. Therefore, several studies have been performed to date in an attempt to elucidate how dysregulation of these compounds affects the integrity of the barrier in skin disorders^11^,^12^, including psoriasis^13^,^14^,^15^. The plethora of sphingolipids that can be derived from the combination of sphingoid base classes (*e.g.,* sphingosine, phytosphingosine), fatty acid types (*e.g.,* hydroxylated, esterified) and fatty acid chain lengths (*e.g.,* C_12:0_, C_16:0_) renders it challenging to simultaneously quantify every potential species^16^. The current study focused on the high abundance plasma-enriched NS-sphingolipid class as well as the important mediators S1P and Spa1P to screen for disease phenotype-specific shifts in circulating sphingolipid levels. Even though differences in mild and severe patients are evident from a clinical diagnosis, circulating markers can be useful to understand potential variations in disease subtypes.

The clinical presentation of psoriasis is highly heterogeneous ranging from minimal essentially cosmetic alterations to widespread generalized disease^1^. In order to maximize potential differences, we selected patients from the polarized groups of mild and severe patients. Severe psoriasis was defined as requiring systemic anti-psoriatic therapy whereas mild disease was defined as a stable phenotype not having been eligible for systemic therapy over the course of at least 10 years following disease onset. All patients were recruited from the same clinic and examined by the same team of dermatologists ensuring a homogenous assessment. In our analysis, no differences in sphingolipids were observed between individuals with mild psoriasis and healthy controls, showing no evidence of circulatory sphingolipid dysregulation in the mild phase of the disease. However, in severe patients, levels of free sphingoid bases were highly increased. These differences in S1P and Spa1P levels between mild and severe psoriasis patients indicate that differences in skin inflammation may be reflected also in other biological compartments.

Both sphinganine and sphingosine are ceramide precursors that are phosphorylated via sphingosine kinases to produce Spa1P and S1P (Supplementary Figure 1). S1P metabolism is associated with diseases that have inflammatory and autoimmune components including multiple sclerosis^17^ and rheumatoid arthritis^18^. In addition, S1P has recently been associated with cardiovascular disease. For example, S1P has been shown to maintain vascular integrity^19^ and the high density lipoprotein (HDL)-associated ApoM-S1P complex has a vasoprotective function in the endothelium^20^. However, high serum S1P levels have also been reported to be predictors of obstructive coronary artery disease^21^. In addition, the ratio of S1P/HDL was found to increase in patients with stable coronary artery disease and following myocardial infarction^22^. Psoriasis patients have been reported to have lower levels of HDL^23^. However, in the current study, HDL levels in the subset of patients with severe psoriasis from which data were available did not evidence significant shifts relative to individuals with mild psoriasis or healthy controls (Supplementary Figure 3). The observed shifts in S1P levels in this subset of patients were the same as observed for the full cohort (Supplementary Figure 3). Taken together, our findings identify potentially important circulatory differences between patients with a constant mild phenotype over many years and severe psoriasis. Importantly, none of the patients included in the mild psoriasis group has progressed to the severe form of the disease in the ten years following sample collection and initial diagnosis. It would be of interest to observe if mild psoriasis patients with high S1P levels are more likely to progress to the severe stage. Several studies indicate a link between psoriasis and increased risk for cardiovascular disease and a recent meta-analysis showed that the association is most likely restricted to patients with severe disease^24^. In our cohort, individuals with severe psoriasis presented significantly higher S1P levels relative to healthy controls, while patients with mild disease did not. This could explain some of the discrepancies observed between studies that have reported that psoriasis is not associated with cardiovascular risk^25^. Interestingly, levels of S1P (and all of the sphingoid bases) were not decreased in severe psoriasis following treatment with Etanercept, suggesting that circulating S1P is associated with severe disease pathology independent of the TNF-α pathway. Taken together, our findings highlight the need to carefully phenotype psoriasis patients and differentiate between patients with a consistent long-term mild phenotype and severe psoriasis in studies relating to S1P.

TNF-α acts through the NF-κβ signaling pathway and its inhibition has been widely demonstrated to be effective against psoriasis^26^. Intracellular S1P has a key role in TNF-α signaling by targeting the TNF receptor-associated factor 2 (TRAF2)^27^. In addition, high concentrations of S1P enhance the response to a suboptimal dose of TNF-α^27^. The fact that high S1P levels are maintained after treatment could also explain the recurrence of the disease once treatment is discontinued. This can also provide insight into whether S1P receptor modulators such as Fingolimod could prove efficacious in treating psoriasis. This theory is further supported by the clinical benefits observed in a recent phase 2 trial for ponesimod, which targets S1P receptors^28^. While the inhibition of NF-κβ and STAT3 has been recently suggested as a therapeutic strategy in psoriasis^29^, Fingolimod has also been found to interfere with the SphK1/S1P/S1P-Receptor 1 feed-forward loop that stimulates the production of IL-6 by the persistent activation of NF-κβ and STAT3^30^. Therefore, it provides a biochemical basis for the potential benefit that patients with severe psoriasis may derive from treatment with S1P receptor modulators.

Ceramide plasma levels were the other primary observed difference between healthy and severe psoriasis groups. Circulating levels of the majority of quantified ceramides (chain lengths 16-24) were higher in severe patients, with the exception of the C_24:0_-species. Interestingly, its behavior was the opposite relative to the shorter fatty acid chain ceramides, with no alterations for C_14:0_ and a decrease in C_12:0_-ceramides. Moreover, a slight non-significant decrease was found in the levels of C_12:0_-sphingomyelins in the severe group (Fig. 2, Supplementary Table 1). Although these decreases were not significant for all compounds, the C_12:0_ species were the only sphingolipids that were affected by anti-TNF-α treatment, which increased their levels. Dysregulation of ceramide levels is known since the early 90’s to occur in psoriatic skin in a ceramide class-dependent manner and with a general increase in the percentage of the NS-class^13^. In addition, several studies have shown that fatty acid chain length composition crucially affects the properties of the skin barrier^5^,^31^,^32^. In particular, very long acyl chain ceramides, like the EOH-ceramide class (which differ from the NS class by the presence of a non-hydroxy fatty acid esterified in the ω-position of the other fatty acid and 4-hydroxysphinganine as the sphingoid base), are crucial for the formation of impermeable lipid lamellae^31^. Specifically, decreases in the EOH-ceramide class are accompanied by increases in the NS-ceramide class in atopic dermatitis, which is associated with increases in trans-epidermal water loss^31^. Additionally, within a ceramide class, fatty acid chain length alterations have been shown to alter the normal function of the skin^5^,^6^,^31^.

Measurements of sphingolipid levels in skin showed that these alterations are also present in lesional skin from severe patients. Global levels of NS-ceramides were drastically increased as has been previously shown^13^. The huge increase in skin ceramides were observed for all fatty acid chain lengths, making it challenging to draw conclusions about their distribution. Nonetheless, increases in C_12:0_-ceramides were less pronounced than for the remainder of the compounds. The analysis of NS-class sphingomyelins showed less marked increases and, interestingly, differences in terms of the fatty acid chain length distribution. These increases were supported by the ceramide synthase (CerS) analyses in the skin. CerS add a fatty acid to sphingosine and sphinganine, and there are six known isoforms in mammals^33^ (Fig. 4b). The highest observed increase was in CerS3, which is the CerS responsible for synthesizing longer acyl chains (Fig. 4b), and is most likely related to the increased levels of C_24:0_-sphingomyelin and C_24:1_-sphingomyelin in skin. CerS4, the most abundant CerS in skin and responsible for the synthesis of C_18_-fatty acids^33^, was also increased. However, the lack of change in levels of C_18_-sphingomyelins in lesional skin may be explained by the concurrent decrease in CerS1, the only CerS that presented lower levels in severe lesional skin. The CerS responsible for synthesis of the C_12_-fatty acid chain length is unknown, but C_12:0_-sphingomyelin was the only compound significantly decreased in non-lesional skin. It needs to be investigated whether this species is synthesized by CerS1, or a yet to be identified CerS.

Although the shift did not reach significance, C_12:0_-sphingomyelin was the only sphingomyelin that decreased in plasma, a trend that was reverted after treatment. There is a paucity of information to date regarding the pathway by which ceramides and sphingomyelins enter the circulation; however, the current study observed an interesting relationship between short-chain sphingolipids in plasma and skin. Moreover, circulating levels of the C_12:0_-ceramide were significantly decreased in severe psoriasis and treatment restored its levels to that of healthy controls. Further studies with chain lengths <12 carbons may be useful for examining this relationship; however, the concentrations of these species will most likely be quite low. There are few reports in the literature of these short chain species, which represent an open area for investigation. It could be expected that TNF-α inhibitor treatment should decrease circulating levels of ceramides by preventing activation of sphingomyelinase SPMD1, which was increased in lesional skin. However, both sphingosine and sphinganine are also important ceramide substrates and treatment did not affect circulating levels. Increases found for sphinganine and dihydroceramides point to an enhanced *de novo* synthesis in the generation of ceramides, which explains why inhibition of sphingomyelinases did not result in decreases in ceramide circulating levels. In skin, increased levels of SPTLC2, one of the subunits of the enzyme responsible for the *de novo* synthesis of sphingolipids, were found in lesional skin, which could explain the increases in the total amount of sphingolipids. Interestingly, alterations in transepidermal water loss, such as occur in psoriasis^14^, have been shown to increase epidermal levels of SPTLC2^34^. Recent studies have also shown the involvement of cytokines in the pathophysiological alteration that occur in psoriatic skin^6^,^35^. For example, Tawada *et al.* reported that interferon-γ modulates the activity of CerS in epidermal models, including decreases of CerS3 activity and suggest the involvement of CerS in atopic dermatitis and psoriasis^6^. Moreover, Danso *et al.* have shown that TNF-α alone or in combination with Th2 cytokines decreases the levels of C_20_ to C_26_ free fatty acids in another skin model^35^. It is thus likely that these decreases are related to the increases in the ceramide equivalents of these fatty acids, via their incorporation into the ceramide backbone. Thus, treatment with anti-TNF-α would act in the skin by restoring the levels and proportions of fatty acids attached to ceramide to a normal profile.

Finally, although the observed increases in UGCG were not reflected in elevated hexosylceramides in the skin, the lactosylceramide derivative did increase (Supplementary Figure 2). Glucosylceramides and UGCG have been implicated as key factors in skin function. For example, the keratinocyte specific Ugcg knockout mouse showed a pronounced desquamation of the stratum corneum and extreme trans-epidermal water loss leading to death^36^. Accordingly, it is possible that the observed UGCG overexpression observed in psoriatic skin may by involved in the disease-associated barrier disruption.

Thus we conclude that even though treatment with TNF-α inhibitors restores skin function in severe psoriasis patients and normalizes total sphingolipid content as well as the fatty acyl chain length profiles in skin, it does not have an impact on overall levels of circulating sphingolipids, except in the case of the low abundance short fatty acid chain C_12:0_-sphingolipids. Most importantly, increased levels of sphingolipids and especially of S1P in severe psoriasis patients requiring biological treatment may potentially be associated with comorbidities (*e.g.,* cardiovascular risk). The observation that circulating S1P levels were not corrected by anti-TNF-α treatment despite improvements in the skin further supports the rationale of targeting S1P receptors as an alternative therapy for severe psoriasis.

## Material and Methods

### Study design and clinical cohort

Healthy donors (n = 32) as well as patients with mild (n = 32) or severe psoriasis (n = 32) were recruited from the Department of Dermatology at the Karolinska University Hospital (Table 1). Severe psoriasis was assessed as requiring systemic therapy for the skin and mild psoriasis as never having required or been eligible for systemic therapy during a 10-year period following disease onset. Samples were collected within a 2-year span. All patients were treated in our department by only 3 physicians in close collaboration ensuring homogenous assessment. Disease was also evaluated by the Psoriasis Area and Severity Index (PASI), which is an established measurement that quantifies the thickness, redness, scaling and distribution of psoriasis lesions^37^. Psoriasis patients had not received systemic immunosuppressive treatment or psoralen+ultraviolet A/solarium/UV for at least 1 month and topical therapy for at least 2 weeks prior to sample collection. This same cohort was previously analyzed by our group in a metabolomics study^9^.

For blood collection, 10 ml of whole blood was collected in EDTA tubes after overnight fasting. Samples were left standing for 1 h before centrifugation at room temperature for 20 min at 1450 *g*. After centrifugation samples were aliquoted and immediately stored at −70 °C until use. Additional plasma samples were taken from the severe psoriasis patients (n = 16) following 12 weeks of Etanercept (Enbrel^®^) treatment (50 mg once per week subcutaneously). The effectiveness of Etanercept treatment was assessed by a significant decrease in the PASI score (before = 13.6 ± 5.5, after = 4.9 ± 3.4; Mann Whitney *P* = 0.00003; Table 1). Four-millimeter punch biopsies were taken from non-lesional and lesional skin from severe psoriasis patients (n = 15) and from non-inflamed, non-irritated skin of healthy individuals (n = 13). A subset of the biopsies (n = 6 severe psoriasis patients and n = 6 healthy individuals) was used for mRNA analysis. The remainder of the biopsies (n = 9 severe psoriasis patients and n = 7 healthy individuals) was screened using the sphingolipid platform. This cohort was distinct from the individuals from which blood samples were collected. The study was approved by the Stockholm Regional Ethics Committee and conducted according to the Declaration of Helsinki’s principles. Signed consent forms were collected from all sample donors.

### LC-MS/MS analysis of sphingolipids in plasma and skin

#### Determination of sphingomyelins, ceramides, dihydroceramides, hexosylceramides and lactosylceramides

200 μL of plasma or ∼15 mg of skin were extracted using a modified Bligh and Dyer procedure^38^. Briefly 10 μL of a set of internal standards containing C_17:0_-Sphingomyelin, C_17:0_-Ceramide, d_17:1_/C_24:1_-Ceramide, C_6:0_-DihydroCeramide, C_8:0_-glucosylceramide and C_17:0_-Lactosylceramide, 1 mL of methanol (CH_3_OH) and 500 μL of chloroform (CHCl_3_) were added to the sample. Samples were then incubated overnight at 48 °C. After bringing samples to room temperature, 150 μL of 1 M KOH in CH_3_OH were added and samples were incubated for 2 h at 37 °C to hydrolyze unwanted species. Then samples were neutralized with glacial acetic acid and 1 mL of CHCl_3_ and 2 mL of H_2_O were added to each sample. After centrifugation for 15 min at 3000 *g*, the lower layer was carefully removed and transferred to a new tube. The remaining pellet was re-extracted by addition of 1 mL of CHCl_3_ and the extracts were then combined and reduced to dryness in a SPEEDVAC^®^ Concentrator from Genevac (Ipswich, UK) and stored at -80 °C until analysis. Extracts were reconstituted in 200 μL of MeOH and filtered using 0.1 μm membrane spin filters and centrifuged for 3.5 min at 3000 *g*. Extracts were then transferred into vials and 7.5 μL of sample were analyzed using a UPLC-MS/MS method on an Acquity UPLC separation module coupled to a Xevo TQ mass spectrometer (Waters, Milford, MS). Chromatographic and mass spectrometry details have been published elsewhere^39^.

#### Determination of sphingoid bases

200 μL of plasma were spiked with 10 μL of internal standard mix containing d_17:1_-Sphingosine, d_17:0_-Sphinganine, d_17:1_-S1P and d_17:1_-Sphinganine1P (Spa1P), and then 1 mL of CH_3_OH and 500 μL of dichloromethane (CH_2_Cl_2_) were added. After overnight incubation at 48 °C, samples were brought up to room temperature, hydrolyzed and neutralized as described above. Samples were then centrifuged for 15 min at 3000 *g*, and the supernatant was removed from the insoluble pellet and transferred to another tube. The pellet was re-extracted by addition of another 1 mL CH_3_OH and 500 uL CH_2_Cl_2_. The solvent fractions were combined and reduced to dryness by SPEEDVAC® Concentrator and stored at −80 °C until analysis. Extracts were reconstituted in 200 μL of 0.5% formic acid in water:MeOH (25:75) and filtered using 0.1 μm membrane spin filters (Merck Millipore, Billerica, MA) and centrifuged for 3.5 min at 5000 *g*. Extracts were then transferred into vials and 7.5 μL were injected and analyzed via UPLC-MS/MS as previously published^39^.

### RNA extraction and quantitative real-time PCR

Total RNA was extracted from skin tissues using miRNeasy Mini kit (Qiagen). Skin biopsies from human were homogenized in liquid nitrogen using a Mikro-Dismembrator U (Braun Biotech) prior to RNA extraction. To quantify mRNAs, 500 ng total RNA was reverse transcribed using the RevertAid First Strand cDNA Synthesis Kit (Fermentas). The mRNAs were quantified by TaqMan gene expression assays (Applied Biosystems). Target gene expression was normalized based on the expression of the internal positive control 18S RNA: 5′-CGGCTACCACATCCAAGGAA-3′ (forward), 5′-GCTGGAATTACCGCGGCT-3′ (reverse), and 5′-FAM-TGCTGGCACCAGACTTGCCCTC-TAMRA-3′ (probe). The following enzymes were screened: ACER1: Alkaline ceramidase 1; ACER3: Alkaline ceramidase 3; CERS1: Ceramide synthase 1; CERS2: Ceramide synthase 2; CERS3: Ceramide synthase 3; CERS4: Ceramide synthase 4; CERS5: Ceramide synthase 5; CERS6: Ceramide synthase 6; GBA: Glucosylceramisase; SGMS1: Sphingomyelin Synthase 1; SGMS2: Sphingomyelin Synthase 2; SGPL1: Sphinganine-1-phosphate aldolase; SMPD1: Sphingomyelin phosphodiesterase 1; SMPD2: Sphingomyelin phosphodiesterase 2; SMPD3: Sphingomyelin phosphodiesterase 3; SPTLC1: Serine Palmitoyl Transferase Long Chain Subunit 1; SPTLC2: Serine Palmitoyl Transferase Long Chain Subunit 2; SPTLC3: Serine Palmitoyl Transferase Long Chain Subunit 3; SPHK1: Sphingosine Kinase 1; SPHK2: Sphingosine Kinase 2; UGCG: UDP-glucose glucosyltransferase; UGT8: 2-hydroxyacylsphingosine 1-beta-galactosyltransferase.

### Statistical analysis

Statistical analysis was conducted using IBM SPSS Statistics Version 22 (SPSS Inc., Chicago, IL, USA) and Graph Pad Prism 5.0 for Windows (GraphPad Software, San Diego, CA, USA). Normality was tested via a Kolmogorov-Smirnov test. One-way ANOVA with Tukey´s post-hoc comparisons and Kruskal-Wallis test with Dunn´s posthoc comparisons were used for multiple group comparisons of normally and non-normally distributed data, respectively. Fisher’s exact test (for categorical variables) and ANOVA (for comparisons of means of continuous variables) were used for the comparison of demographic and exposure variables between the groups in Table 1. Paired samples were compared using a two-sided Wilcoxon signed-rank test. A two tailed *P* value <0.05 was considered as significant.

## Additional Information

**How to cite this article**: Checa, A. *et al.* Circulating levels of sphingosine-1-phosphate are elevated in severe, but not mild psoriasis and are unresponsive to anti-TNF-α treatment. *Sci. Rep.* **5**, 12017; doi: 10.1038/srep12017 (2015).

## Supplementary Material

Supplementary Information

## Figures and Tables

**Table 1:** Demographic characteristics of the clinical cohort (a) healthy *vs.* mild and severe psoriasis patients and (b) severe psoriasis patients before and after anti-TNF-α treatment.

**a)**	**Healthy controls**	**Mild psoriasis**	**Severe psoriasis**	**One-way ANOVA P-value**
N	32	32	32	
Gender (Female)	16/32	16/32	16/32	1.000
Age (years)	51.5 ± 13.6	50.6 ± 17.1	55.9 ± 12.1	0.298
BMI^a^	25.1 ± 3.7	25.1 ± 4.7	27.4 ± 4.5	0.050
Cholesterol (nmol/L)	5.1 ± 0.8	5.0 ± 0.9	5.1 ± 1.0	0.869
Triglycerides (nmol/L)	1.1 ± 0.6	1.1 ± 0.8	1.2 ± 0.5	0.583
PASI score^b^	N.A.	1.5 ± 0.9	15.1 ± 6.3	2 × 10^−13^
**b)**	**Before treatment**	**After treatment**	**Student's t-test P-value**	
N	16	16		
Gender (Female)	8/16	8/16	1.000	
Age (years)	53.6 ± 13.7	53.6 ± 13.7	1.000	
BMI	27.3 ± 4.8	27.2 ± 4.6	1.000	
Cholesterol (nmol/L)	5.3 ± 0.6	6.6 ± 4.1	0.412	
Triglycerides (nmol/L)	1.0 ± 0.4	1.7 ± 0.9	0.337	
PASI score	13.6 ± 4.5	4.9 ± 3.4	2 × 10^-5^	

^a^BMI: Body mass index.

^b^PASI: Psoriasis area and severity index^37^. Comparison is only performed for the mild and severe psoriasis groups. N.A.: Not applicable.
