# Supplementary material for: Circulating levels of sphingosine-1-phosphate are elevated in severe, but not mild psoriasis and are unresponsive to anti-TNF-α treatment
Source: Sci Rep. 2015 Jul 15;5:12017. doi: 10.1038/srep12017 (PMC4502512; doi:10.1038/srep12017)
Supplement: Supplementary Information [file srep12017-s1.pdf]

Supplementary material

Circulating levels of sphingosine-1-phosphate are elevated in severe, but not mild psoriasis and are unresponsive to anti-TNF-α treatment

Antonio Checa[1], Ning Xu[2], Daniel G. Sar[1], Jesper Z. Haeggström[1], Mona Ståhle[2**], Craig E. Wheelock[1**]

[1]Department of Medical Biochemistry and Biophysics, Division of Physiological Chemistry 2, Karolinska Institutet, SE-17177, Stockholm, Sweden.

[2]Dermatology Unit, Department of Medicine, Karolinska Institutet SE-17176, Stockholm, Sweden

**Corresponding authors

Supplementary Table 1. Plasma sphingolipid concentrations measured for the different groups

Compound	Concentration (nM)[a]			p-value [b,c]	
	HC (n = 32)	MP (n = 32)	SP (n = 32)	(HC vs SP)	(MP vs SP)
**Sph (d18:1)**	29.1 (9.3)	30.2 (10.1)	47.6 (12.8)	7.7e-9	2.3e-8
**Spa (d18:0)**	7.1 (2.4)	7.5 (3.2)	15.6 (8.4)	2.5e-8	9.6e-8
**S1P (d18:1)**	718.9 (150.4)	663.3 (196.7)	963.6 (178.2)	7.9e-7	7.8e-9
**Spa1P (d18:0)**	329.6 (78.6)	306.9 (114.0)	448.3 (117.1)	5.2e-5	1.5e-6
**SM 12:0**	289.7 (87.2)	277.9 (69.5)	253.2 (93.8)	NS	NS
**SM 16:0**	38602.8 (8756.0)	36986.1 (6494.6)	40860.9 (8769.5)	NS	NS
**SM 18:1**	6850.9 (1121.7)	6652.5 (1002.2)	7418.9 (1901.8)	NS	NS
**SM 18:0**	14739.1 (2207.5)	14705.2 (2085.5)	16536.1 (3106.5)	1.4e-2	1.2e-2
**SM 24:1**	30119.4 (5676.2)	29136.5 (4423.4)	31338.1 (6292.5)	NS	NS
**SM 24:0**	14181.6 (3533.1)	13728.9 (3126.4)	14883.9 (3367.6)	NS	NS
**Cer 12:0**	1.3 (1.0)	0.9 (0.8)	0.8 (0.8)	4.8e-2	NS
**Cer 14:0**	13.5 (4.1)	12.9 (3.9)	13.7 (6.5)	NS	NS
**Cer 16:0**	220.0 (51.9)	218.9 (48.8)	260.8 (75.1)	3.0e-2	2.8e-2
**Cer 18:1**	14.5 (2.6)	14.7 (2.7)	16.4 (4.4)	NS	NS
**Cer 18:0**	117.7 (41.0)	121.9 (39.5)	159.0 (55.4)	3.0e-3	1.2e-2
**Cer 20:0**	93.6 (26.4)	100.1 (28.3)	116.5 (25.1)	6.0e-3	4.2e-2
**Cer 22:0**	1465.1 (522.1)	1483.3 (493.7)	1826.9 (635.1)	3.9e-2	NS
**Cer 24:1**	1937.0 (525.7)	2057.7 (624.9)	2352.6 (631.0)	4.1e-2	NS
**Cer 24:0**	2746.7 (889.4)	2735.6 (1041.6)	2949.6 (926.4)	NS	NS
**DhCer 16:0**	37.0 (8.5)	38.2 (12.1)	45.3 (11.9)	9.0e-3	3.1e-2
**DhCer 24:0**	5262.5 (2414.9)	5293.8 (2485.7)	6975.7 (3173.5)	NS	NS
**HexCer 12:0**	1.0 (0.7)	1.0 (0.8)	0.9 (0.8)	NS	NS
**HexCer 16:0**	373.6 (109.3)	386.1 (124.3)	396.0 (112.7)	NS	NS
**HexCer 18:1**	12.8 (2.0)	12.7 (2.0)	14.0 (2.8)	NS	NS
**HexCer 18:0**	24.8 (8.8)	31.1 (18.3)	28.1 (10.3)	NS	NS

**HexCer 24:1**	745.6 (166.5)	823.6 (218.6)	874.9 (313.5)	NS	NS
**LacCer 12:0**	17.1 (8.2)	17.1 (12.8)	14.8 (8.5)	NS	NS
**LacCer 16:0**	3046.6 (759.5)	3150.7 (888.5)	3288.4 (995.1)	NS	NS
**LacCer 24:1**	875.0 (299.3)	926.0 (298.8)	911.7 (294.9)	NS	NS
**LacCer 24:0**	119.0 (38.6)	125.0 (38.9)	130.8 (38.0)	NS	NS

[a]Data are presented as mean(SD). Abbreviations: HC=Healthy Control; MP=Mild Psoriasis; SP=Severe Psoriasis; Sph=Sphingosine; Spa=Sphinganine; SM=Sphingomyelin; Cer=Ceramide; DhCer=Dihydroceramide; HexCer=Hexosylceramide; LacCer=Lactosylceramide; NS=Not significant via ANOVA or Kruskal-Wallis test.

[b]The normality of the distribution was assessed by the Kolmogorov-Smirnov test. One-way ANOVA with Tukey´s post-hoc comparisons and Kruskal-Wallis test with Dunn´s posthoc comparisons are provided for normally and non-normally distributed data according to the Kolmorogov-Smirnov test, respectively.

[c]No differences were found for HC *vs*. MP group comparisons.

Supplementary Table 2. Changes in plasma sphingolipid levels in individuals with severe psoriasis following Etanercept treatment (n=16).[a]

	Fold change (After/Before)	p-value
**Sph (d18:1)**	1.03	NS
**Spa (d18:0)**	0.97	NS
**S1P (d18:1)**	1.04	NS
**Spa1P (d18:0)**	1.04	NS
**SM 12:0**	1.21	<0.01
**SM 16:0**	1.03	NS
**SM 18:1**	0.96	NS
**SM 18:0**	0.97	NS
**SM 24:1**	1.01	NS
**SM 24:0**	1.03	NS
**Cer 12:0**	1.32	<0.05
**Cer 14:0**	1.02	NS
**Cer 16:0**	0.96	NS
**Cer 18:1**	0.98	NS
**Cer 18:0**	0.89	NS
**Cer 20:0**	1.02	NS
**Cer 22:0**	1.00	NS
**Cer 24:1**	0.98	NS
**Cer 24:0**	1.10	NS
**HexCer 12:0**	1.31	NS
**HexCer 16:0**	1.08	NS
**HexCer 18:1**	1.02	NS
**HexCer 18:0**	1.05	NS
**HexCer 24:1**	1.05	NS
**LacCer 12:0**	1.15	<0.05
**LacCer 16:0**	0.99	NS
**LacCer 24:1**	1.01	NS
**LacCer 24:0**	1.00	NS
**DhCer 16:0**	1.02	NS
**DhCer 24:0**	1.09	NS

[a]Abbreviations: Cer=Ceramide; SM=Sphingomyelin; HexCer=Hexosylceramide; LacCer=Lactosylceramide; DhCer=Dihydroceramide. Significance levels were determined using a two-sided Wilcoxon signed-rank test. NS=Not-significant.

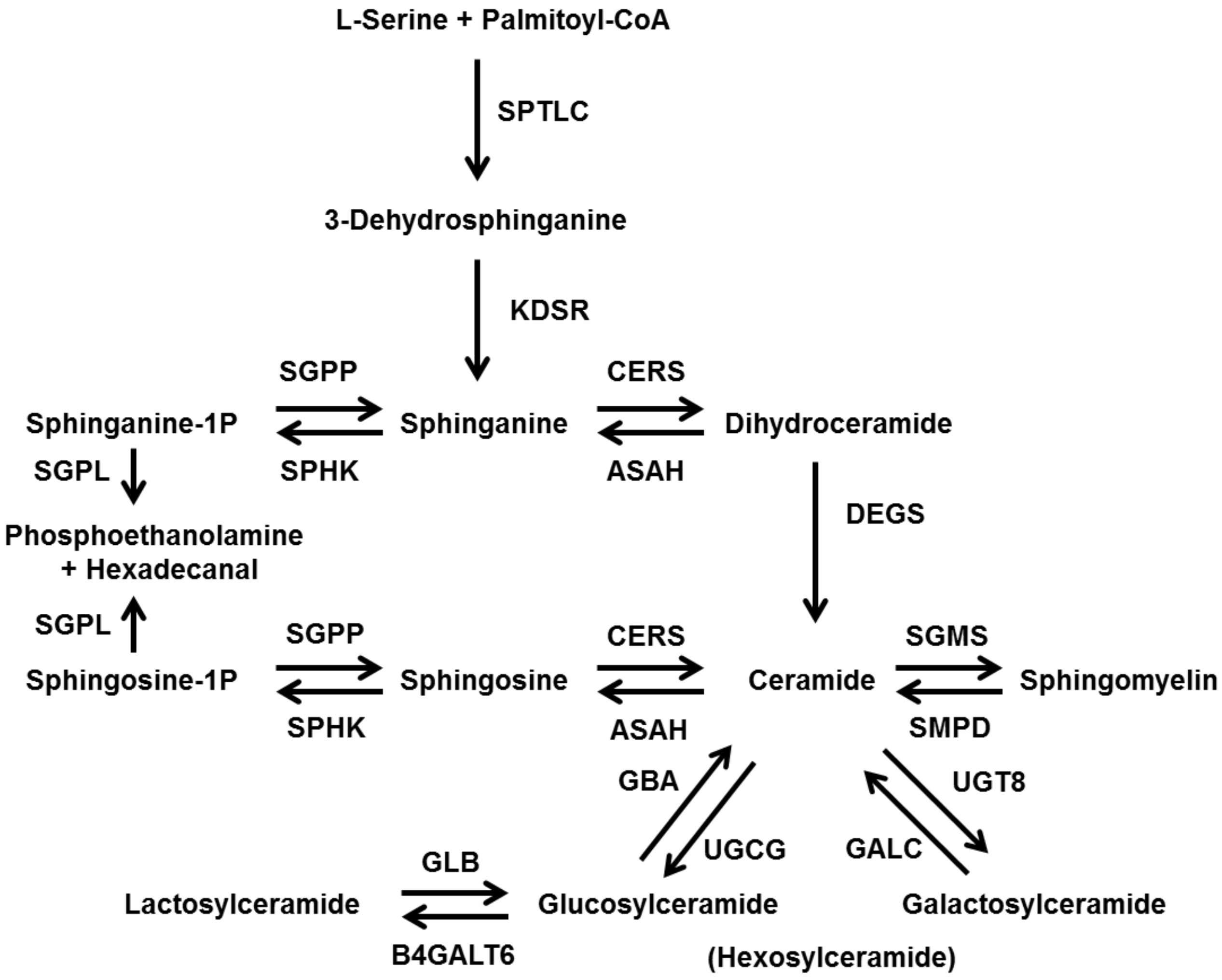

Supplementary Figure 1. Scheme of the sphingolipid pathway including sphingolipids and their related enzymes. ASAH: N-acylsphingosine amidohydrolase (ceramidase); B4GALT6: beta-1,2-galactosyltransferase 6; CERS: Ceramide synthase; DEGS: Sphingolipid delta 4-desaturase; GALC: Galactosylceramidase; GBA: Glucosylceramisase; GLB: Beta-galactoside; KDSR: 3-dehydrosphinganine reductase; SGPL: Sphinganine-1-phosphate aldolase; SGPP: Sphingosine-1-phosphate phosphatase; SGMS: Sphingomyelin Synthase; SMPD: Sphingomyelin phosphodiesterase; SPTLC: Serine Palmitoyl Transferase; SPHK: Sphingosine Kinase; UGCG: UDP-glucose glucosyltransferase; UGT8: 2-hydroxyacylsphingosine 1-beta-galactosyltransferase.

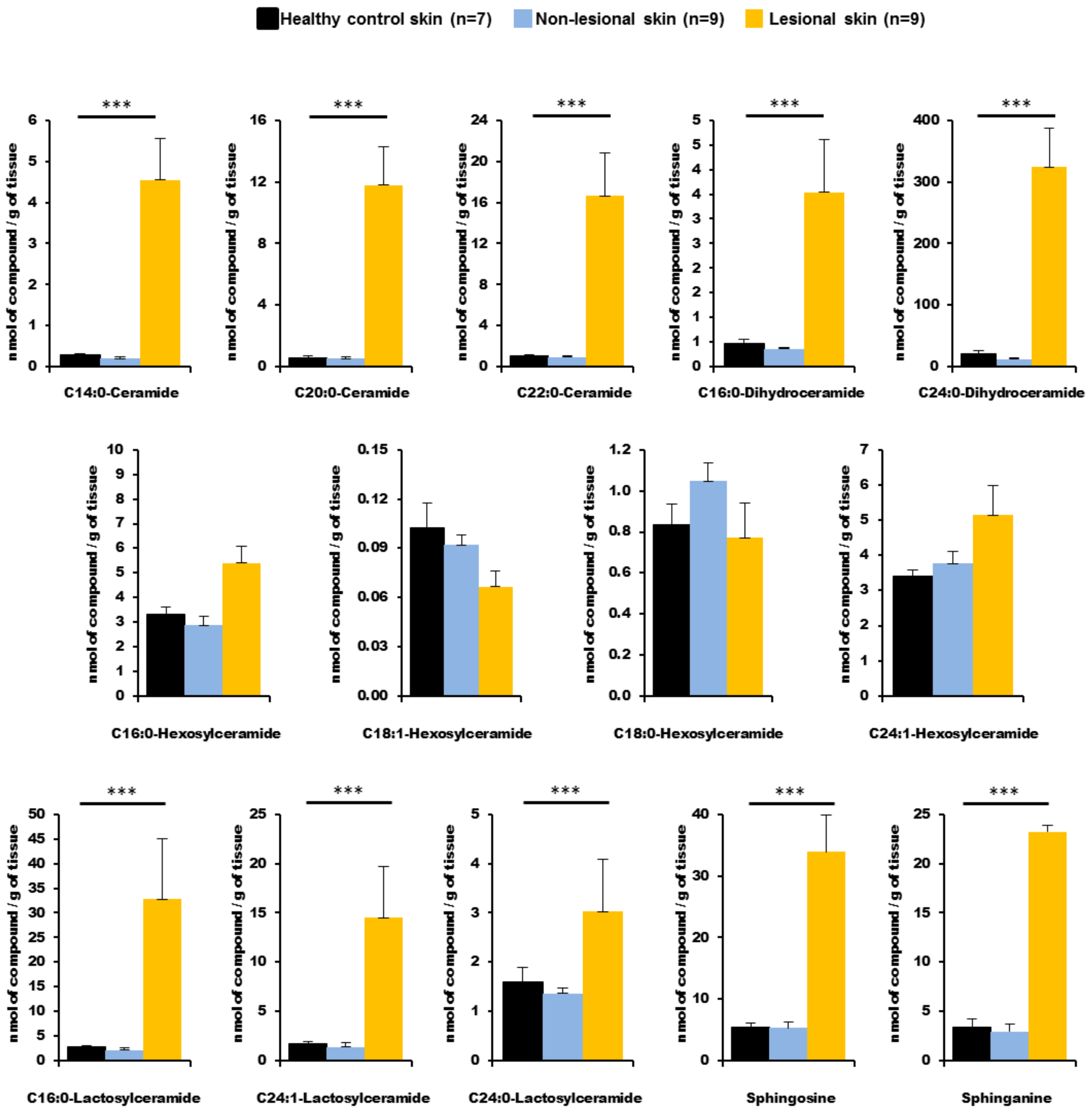

Supplementary Figure 2. Levels of the sphingolipids not shown in Figure 3 in lesional and non-lesional skin from severe psoriasis patients relative to healthy controls. Data are presented as mean±SEM. Statistical significance was determined by a Kruskal-Wallis with Dunn´s post-hoc correction comparing severe groups to the healthy control group. **P*<0.05; ***P*<0.01; ****P*<0.001.

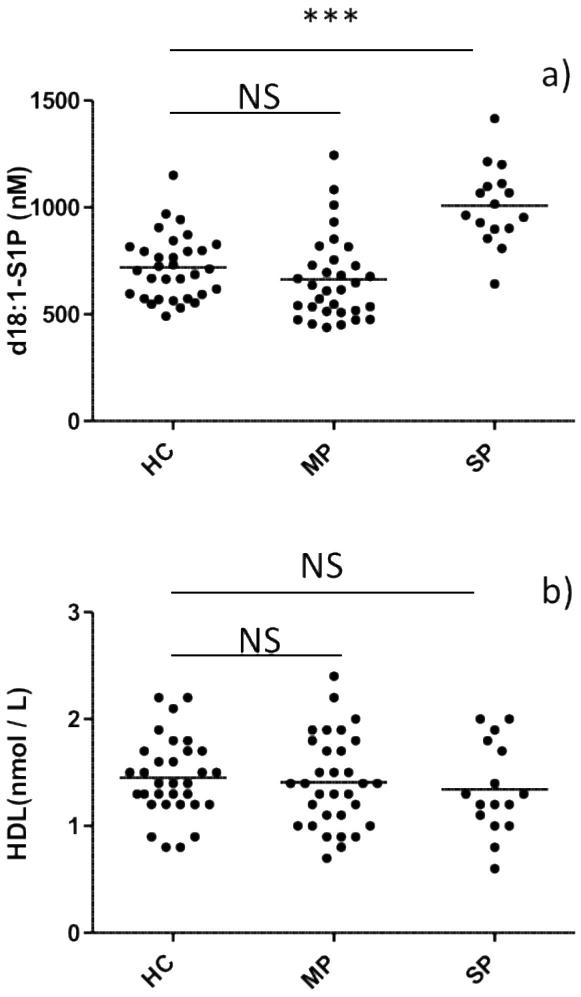

Supplementary Figure 3. Plasma levels of S1P and HDL in the different groups. Between group comparison of levels of **a)** S1P and **b)** HDL for patients with HDL data available (HC [n=32], MP [n=32], SP [n=16]). Statistical significance was determined by one-way ANOVA with Dunnet´s post-hoc comparison. Abbreviations: HC=Healthy controls; MP=Mild psoriasis patients; SP=Severe psoriasis patients; NS=Not significant; ****P*< 0.001.
